# Measurement Invariance of a Classroom Engagement Measure among Academically At-Risk Students

**DOI:** 10.3389/fpsyg.2017.02345

**Published:** 2018-01-12

**Authors:** Ryan Glaman, Qi Chen

**Affiliations:** ^1^Department of Educational Leadership and Policy Studies, Tarleton State University, Stephenville, TX, United States; ^2^Department of Educational Psychology, University of North Texas, Denton, TX, United States

**Keywords:** engagement, longitudinal data analysis, measurement invariance, multigroup comparison, confirmatory factor analysis

## Abstract

The current study investigated the measurement invariance of a classroom engagement measure across time points, genders, and ethnicities using a sample of 523 academically at-risk students across grades 7 through 9; this measure was based on Skinner et al.'s (1990) original engagement measure. The engagement measure was comprised of 16 items, yielding three factors: Behavioral Engagement, Behavioral Disaffection, and Emotional Engagement. Configural, metric, and scalar invariance held across the three time points, as did invariance of factor covariances and means, indicating that scores have a similar meaning across all 3 years. The engagement measure also featured adequate configural, metric, and scalar invariance, and invariance of factor covariances and means across genders and ethnicities. These findings suggest the measure is appropriate for investigating substantive hypotheses regarding classroom engagement across different grade levels, genders, and ethnicities. In summary, the current results indicate this measure of classroom engagement is suitable for testing hypotheses regarding group differences in engagement across grade levels, genders, and ethnicities. Researchers may also use this measure to examine relationships between the engagement factors and other important academic outcomes. Limitations of the current study, such as certain caveats regarding convergent validity and internal consistency, are also discussed.

## Introduction

Students' engagement in the classroom is strongly related to academic performance outcomes such as reading performance (Lee, 2014; Lutz Klauda and Guthrie, 2015), mathematics performance (Rimm-Kaufman et al., 2015), and general academic performance (Skinner et al., 1990; Chen et al., 2010). Engagement is also related to other academic variables such as student-teacher relationship quality (Wu et al., 2010) and reading motivation (Lutz Klauda and Guthrie, 2015). Furthermore, failing to engage in the classroom is related to various negative outcomes such as delinquency, substance abuse, and dropout rates (Wang and Fredricks, 2014). Because engagement has relationships with several important academic variables, it is important to consider in both educational research and practice.

One issue with academic engagement as a construct is that there are differences across research studies in terms of its measurement and theoretical definition. For example, some researchers conceptualize engagement as a three-factor construct consisting of behavioral, emotional, and cognitive components (e.g., Burch et al., 2015; Sinatra et al., 2015). Other researchers suggest engagement includes not only behavioral and emotional engagement, but an engagement vs. disaffection component as well; therefore, according to some researchers, engagement is conceptualized as a four-factor construct that includes behavioral engagement, behavioral disaffection, emotional engagement, and emotional disaffection (e.g., Skinner et al., 2008, 2009). Furthermore, engagement may be measured in general, as described above, or it may be domain-specific, such as when measuring reading (Lutz Klauda and Guthrie, 2015) or mathematics engagement (Rimm-Kaufman et al., 2015).

Classroom engagement's theoretical diversity is accompanied by diversity in its measurement as well. Some engagement measures are designed to encapsulate its behavioral, emotional, and cognitive components (Wang and Fredricks, 2014), whereas others attempt to capture emotional and behavioral engagement and disaffection (Skinner et al., 2008). Furthermore, there can also be diversity within a given theoretical perspective; for example, various studies examining emotional and social engagement and disaffection tend to use similar, but slightly different versions of an engagement measure (e.g., Skinner et al., 1998, 2008; Wu et al., 2010).

Despite the complexities with theoretically defining and measuring engagement, the goal of the current study was to use measurement invariance (MI) testing procedures to examine the psychometric properties of a measure of classroom engagement. The measure of interest has been used in empirical research (Chen et al., 2010) and is based on Skinner et al. (1998) measure. This particular measure was chosen because it taps into dimensions of behavioral and emotional engagement and disaffection, theoretical constructs that are well-established in the literature (Skinner et al., 1990, 1998, 2008, 2009) and that predict important outcomes such as academic performance.

### Measurement invariance

Generally speaking, MI testing procedures examine the equivalence of a test's measurement across distinct groups of individuals such as genders or ethnicities. Measurement invariance can be tested using a series of multigroup confirmatory factor analyses (CFAs) that impose increasingly stringent criteria on the model (Cheung and Rensvold, 2002; Millsap, 2011). The first criterion is configural invariance, in which the groups have the same pattern of factor coefficients and “zero-loadings” on the factors; that is, the groups conceptualize the concepts the same way (Vandenberg and Lance, 2000; Cheung and Rensvold, 2002). The next criterion is metric invariance, which examines the equality of the factor loadings across groups. Metric invariance is an important prerequisite for meaningful cross-group comparisons. The third criterion, scalar invariance, refers to the equality of item intercepts across groups. Scalar invariance indicates the latent constructs are measured on the same scale across groups and is necessary for comparing groups' factor means. MI can be assessed not only at the item-level, but at the construct-level as well; for example, the invariance of latent factor means or covariances may be examined across groups. These two tests of MI are typically based on theory and may be used to address substantive research questions (Cheung and Rensvold, 2002).

In general, a test must possess MI across groups in order to make cross-group comparisons on the constructs being measured. While MI can be assessed cross-sectionally, it can also be examined using data gathered longitudinally over multiple occasions (Vandenberg and Lance, 2000). Procedurally, longitudinal MI can be examined using either a multisample approach (i.e., similar to examining cross-sectional MI) or by using an augmented covariance matrix as input (Vandenberg and Lance, 2000). For the current study, the former approach was chosen to avoid the shortcomings associated with the augmented covariance matrix approach, such as increased likelihood of non-convergence and generally worse model fit.

Existing psychometric literature has examined MI for various types of engagement measures, but none have explored the measure derived from Skinner et al. (1998) conceptualization. Some studies have examined MI of measures that include elements of cognitive, affective, and behavioral engagement (e.g., Glanville and Wildhagen, 2007; Wang et al., 2011), observing that these measures are largely invariant across ethnicities and genders. Other studies have tested MI for engagement measures featuring more complex factor structures. For example, Bradshaw et al. (2014) examined the MI of Maryland's Safe and Supportive Schools Initiative survey, which features an engagement measure including six factors: teacher connectedness, student connectedness, academic engagement, whole-school connectedness, culture of equity and fairness, and parent engagement; the authors found this measure was invariant across genders, ethnicities, and grade levels. Other studies have also tested the MI of the Motivation and Engagement Scale, which features five engagement factors: persistence, planning, task management, disengagement, and self-handicapping. Marsh et al. (2011) found this measure was invariant across genders and time points, whereas Martin et al. (2015) showed it was invariant across samples from different countries.

### Purpose of the current study

Existing psychometric literature on measures of student engagement has yet to examine a measure featuring the theoretical conceptualization described by Skinner et al. (1998), which includes: behavioral engagement, behavioral disaffection, emotional engagement, and emotional disaffection. Therefore, the goal of the current study was to investigate the MI of such an engagement measure longitudinally across students in grades 7 through 9 as well as across ethnicity and gender.

## Method

### Participants

Participants included 523 students attending one of three school districts in Texas (one urban and two small cities). These participants were selected because they were part of a larger longitudinal study investigating the impact of grade retention on academic achievement among at-risk students, in which classroom engagement was also a variable of interest. Participants were recruited across two sequential cohorts in first grade during the fall of 2001 and 2002. Children were eligible to participate in the longitudinal study if they scored below the median score on a state-approved, district-administered measure of literacy, spoke either English or Spanish, were not receiving special education services, and had not previously been retained in first grade. School records identified 1,374 students as being eligible to participate. Because teachers distributed consent forms to parents via children's weekly folders, the exact number of parents who received the consent forms could not be determined. Small gifts to children and the opportunity to win a larger prize in a random drawing were instrumental in obtaining 1,200 returned consent forms, of which 784 parents (65%) provided consent. Analyses on a broad array of archival variables including performance on the district-administered test of literacy, age, gender, ethnicity, eligibility for free or reduced-price lunch, bilingual class placement, cohort, and school context variables (i.e., ethnic composition and percentage of economically disadvantaged students), did not indicate any differences between children with and without consent.

Of these 784 participants, 523 (66.7%) met the inclusion criteria for participation in the current study: they had engagement data from at least one assessment wave, and they were still registered as active in the study at year 9. The sample was 45.0% female and 55.0% male, and its ethnic composition was 37.2% Hispanic, 33.5% White, 25.5% Black, and 3.8% other. A cross-tabulation of ethnic and gender groups is shown in Table 1. The results of a chi-square test showed that each of these groups were represented equally within the sample, $\chi_{\left(5\right)}^{2}$ = 6.282, *p* = 0.280, Cramer's *V* = 0.110.

**Table 1:** Cross-tabulation of participant demographic frequencies by ethnicity and gender.

**Gender**	**Ethnicity**				**Row Total**
	**Hispanic**	**Black**	**White**	**Other**	
Female	91	63	70	11	235
Male	104	71	104	9	288
Column total	195	134	174	20	523

Based on attrition analyses, the 523 students in the current sample did not differ from the 261 students who did not complete the study in terms of most demographic variables including: ethnicity, age, socioeconomic status, reading achievement scores based on the Woodcock-Johnson III Broad Reading test (Woodcock et al., 2001), and base-year engagement scores. However, a larger proportion of males remained active in the study than females [$\chi_{\left(1\right)}^{2}$ = 3.988, *p* = 0.046, Cramer's *V* = 0.071], a smaller proportion of bilingual students remained active in the study [$\chi_{\left(1\right)}^{2}$ = 4.615, *p* = 0.032, Cramer's *V* = 0.077], active students scored slightly higher on the Woodcock-Johnson III Broad Math test than inactive students [*F*_(1, 754)_ = 6.724, *p* = 0.010, η^2^ = 0.009], and a larger proportion of students whose parents obtained a high school diploma remained active in the study whereas a larger proportion of students whose parents obtained a graduate-level degree dropped out of the study [$\chi_{\left(4\right)}^{2}$ = 11.173, *p* = 0.025, Cramer's *V* = 0.119]. However, because the effect sizes for these differences between active and inactive students were small, it was assumed that there were no practical differences between students who dropped out of the study and those who did not.

This study was carried out in accordance with the recommendations of the Institutional Review Board of Texas A&M University with written informed consent from all participants. All participants gave written informed consent in accordance with the Declaration of Helsinki. The protocol was approved by the Institutional Review Board of Texas A&M University.

### Design overview

Assessments were conducted annually for 9 years, beginning when participants were in the first grade (year 1). Student-report classroom engagement was assessed at years 4, 7, 8, and 9 (these correspond to grades 4, 7, 8, and 9, respectively). However, substantive research suggests developmental differences in engagement exist; that is, students' classroom engagement is likely to shift dramatically between elementary and middle school (Skinner et al., 2008, 2009; Wang et al., 2014). These changes in classroom engagement have been attributed to younger children being developmentally different than young adults in terms their learning strategies, self-regulation, and other factors relevant to classroom engagement (Fredricks and McColskey, 2012; Sinatra et al., 2015). Because fourth grade students are substantially developmentally different from seventh, eighth, and ninth grade students in terms of the cognitive attributes associated with engagement, it would be inappropriate to directly compare those two age groups. Therefore, the year 4 assessment wave was dropped, and only data from years 7, 8, and 9 were included in the MI analyses.

### Engagement measure

Student engagement was measured using a student-report, 18-item scale based on Skinner et al. (1998) original measure. Both English and Spanish versions of the measure were available, and students completed the measure using the language they were more proficient in; ~3.63% of students completed the engagement measure in Spanish at year 7, ~2.68% of students completed the measure in Spanish at year 8, and ~1.91% of students completed the measure in Spanish at year 9. Students indicated how true each item was in describing them using a 1–4 scale (1 = “not at all true,” 4 = “very true”). Previous empirical research using this measure of student classroom engagement suggests that it contains three latent factors, Behavioral Engagement, Behavioral Disaffection, and Emotional Engagement, and that one item should be removed from the measure due to low factor loadings (Chen et al., 2010). Example Behavioral Engagement scale items include “When I am in class, I work as hard as I can,” and “I try to learn as much as I can about my school subjects.” Example Behavioral Disaffection scale items include “When I am in class, I just act like I am working,” (reverse scored) and “When I am in class, I just try to look busy” (reverse scored). Example Emotional Engagement scale items include “When I am in class, I feel angry” (reverse scored), and “When I am in class, I feel happy.” The internal consistency reliabilities for the current sample across all three time points are shown in Table 2. Because dropping an item assessing feeling anxious in class increased the internal consistency of the Emotional Engagement composite scale, one item was dropped, reducing the total number of items in the measure to 16. Overall, the Behavioral Engagement and Behavioral Disaffection scales featured adequate internal consistency reliability across all three measurement occasions according to generally accepted standards (Henson, 2001), but the Emotional Engagement scale did not.

**Table 2:** Cronbach's alpha reliabilities of the engagement scales across 3 years.

	**Behavioral Engagement (7 Items)**	**Behavioral Disaffection (6 Items)**	**Emotional Engagement (3 Items)**
Year 7	0.810	0.810	0.552
Year 8	0.819	0.795	0.517
Year 9	0.825	0.810	0.572

### Data analysis overview

A series of MI analyses were conducted to examine the longitudinal stability of the engagement measure's structure across 3 years; the configural, metric, and scalar invariance assumptions, as well as invariance of factor covariances and means, were sequentially tested using a CFA framework. Before conducting the MI analyses, the normality of the engagement item responses was examined across the 3 years. All the skewness and kurtosis statistics were within the acceptable range (skewness within ±3.00 and kurtosis within ±8.00; Kline, 2010), indicating all the study variables were normally distributed. We tested the three-factor CFA model from previous research as described above (Chen et al., 2010) with data from the three assessment waves. Model chi-square test statistics, along with Hu and Bentler's (1999) commonly used model fit criteria, were used to provide evidence of adequate model fit. Modification indices were used to provide statistical evidence for the unknown underlying relationships among items. An examination of modification indices indicated that that three pairs of correlated items had consistently large modification indices across all 3 years (i.e., modification index ≥20). Items 2 and 6 both involved concentrating on doing class work. Items 9 and 10 both involved trying to look busy during class. Lastly, items 15 and 17 both involved thinking about non-class-related things during class time. As researchers recommend that use of modification indices have substantive justification (Byrne, 1998; Kline, 2010), the original three-factor CFA was modified to include these three pairs of correlated items due to the pairs' content similarity. This revised three-factor CFA demonstrated an adequate model goodness-of-fit across all three assessment waves, average comparative fit index ($\bar{CFI}$) = 0.952, average Tucker-Lewis index ($\bar{TLI}$) = 0.941, average root-mean-square error of approximation ($\bar{RMSEA}$) = 0.049, average standardized root mean square residual ($\bar{SRMR}$) = 0.050. Fit indices for the three individual assessment waves are shown in Table 3.

**Table 3:** CFA model fit indices across all three measurement occasions.

**Year**	**CFI**	**TLI**	**RMSEA**	**SRMR**
7	0.955	0.945	0.048	0.051
8	0.956	0.946	0.047	0.046
9	0.945	0.933	0.053	0.053

Additionally, the engagement factors' convergent and discriminant validity were assessed across all three time points by examining the standardized factor pattern/structure coefficients, average variance extracted (AVE) for each factor, and factor correlations. Standardized factor pattern/structure coefficients and associated standard errors across all 3 years are shown in Table 4 and factor AVEs, correlations, and squared correlations are all shown in Table 5. Kline (2010) suggested that standardized factor pattern coefficients of at least 0.70 indicate good convergent validity. While the majority of items across the engagement subscales met or came close to meeting this threshold (see Table 4), certain items were problematic across time points, specifically items 13, 14, 15, 16, and 17. Regarding discriminant validity, although the squared factor correlations are relatively high compared to the AVEs in several cases (see Table 5), moderately high correlations between these engagement factors have also been observed in prior research (e.g., Skinner et al., 2009), suggesting that the correlations in the present study are consistent with existing theory. Furthermore, Kline (2010) suggested that as long as factor correlations are not excessively high (i.e., ≥0.90 in absolute value), that is evidence of adequate discriminant validity. Therefore, despite relatively low pattern coefficients for select items and high factor correlations in some cases, the current three-factor model demonstrated moderate convergent and discriminant validity across all three time points.

**Table 4:** Standardized factor pattern/structure coefficients (and standard errors) for the CFA model across 3 years.

**Item**	**Year 7**	**Year 8**	**Year 9**
**BehEng**			
Item 1	0.668 (0.047)	0.669 (0.046)	0.663 (0.039)
Item 2	0.705 (0.031)	0.731 (0.030)	0.698 (0.030)
Item 5	0.783 (0.028)	0.814 (0.025)	0.823 (0.024)
Item 6	0.738 (0.029)	0.756 (0.028)	0.759 (0.028)
Item 7	0.725 (0.033)	0.721 (0.035)	0.723 (0.030)
Item 13	0.509 (0.048)	0.503 (0.044)	0.413 (0.047)
Item 14	0.430 (0.041)	0.419 (0.041)	0.479 (0.040)
**BehDis**			
Item 9^*^	0.656 (0.041)	0.729 (0.034)	0.697 (0.036)
Item 10^*^	0.692 (0.038)	0.702 (0.041)	0.749 (0.029)
Item 11^*^	0.668 (0.034)	0.663 (0.037)	0.735 (0.033)
Item 15^*^	0.566 (0.037)	0.554 (0.033)	0.604 (0.033)
Item 17^*^	0.497 (0.037)	0.468 (0.041)	0.498 (0.044)
Item 18^*^	0.786 (0.029)	0.703 (0.047)	0.632 (0.042)
**EmoEng**			
Item 8^*^	0.658 (0.056)	0.557 (0.065)	0.722 (0.048)
Item 12^*^	0.682 (0.056)	0.601 (0.063)	0.632 (0.052)
Item 16	0.438 (0.066)	0.534 (0.056)	0.446 (0.059)

* *Denotes reverse-scored items. BehEng, Behavioral Engagement factor; BehDis, Behavioral Disaffection factor; EmoEng, Emotional Engagement factor. Items 3 and 4 are absent from this table because those two items were removed from the measure. Standard errors are shown in parentheses. All pattern coefficients were statistically significant at the p < 0.001 level*.

**Table 5:** Discriminant validity analyses for the engagement measure CFA model across years 7, 8, and 9.

	**Average variance explained**	**Factor correlations (correlations squared)**	
		**BehEng**	**BehDis**
**YEAR 7**			
BehEng	0.439	–	
BehDis	0.423	0.819 (0.671)	–
EmoEng	0.363	0.548 (0.300)	0.746 (0.557)
**YEAR 8**			
BehEng	0.458	–	
BehDis	0.414	0.766 (0.587)	–
EmoEng	0.319	0.711 (0.506)	0.700 (0.490)
**YEAR 9**			
BehEng	0.443	–	
BehDis	0.443	0.794 (0.630)	–
EmoEng	0.373	0.475 (0.226)	0.685 (0.469)

*BehEng, Behavioral Engagement factor; BehDis, Behavioral Disaffection factor; EmoEng, Emotional Engagement factor. All correlations were statistically significant at the p < 0.001 level*.

The CFA structure described above was used as the factor structure in testing the longitudinal MI of the engagement measure across the 3 years. To do so, we followed a procedure recommended by Millsap (2011) and employed by previous MI researchers (e.g., Wu and Hughes, 2015). First, we examined the configural invariance of the measurement structures across the three time points. Next, we tested the metric invariance of items' factor loadings by comparing the model for a given year with the model of the previous year(s). This procedure has two advantages: (a) we can know if the parameters remain the same across the 3 years, and (b) we can detect at which years items become non-invariant. For example, first, we tested the metric invariance between year 7 and year 8 (Λ_7_ = Λ_8_), allowing the factor loadings for year 9 to be freely estimated. Then, we tested the metric invariance between all 3 years (Λ_7_ = Λ_8_ = Λ_9_). After confirming the metric invariance assumption, we tested the scalar invariance assumption, the invariance of factor covariances, and the invariance of factor means using the same procedure.

To evaluate the longitudinal MI and compare the fit of the individual models, we used the chi-square difference test (Δχ^2^; Kline, 2010). However, because the chi-square test is highly sensitive to sample size, we also examined other overall and incremental indicators of model fit. We examined several overall indicators of model fit, such as the CFI, Tucker Lewis Index (TLI), root mean square error of approximation (RMSEA) and SRMR. Hu and Bentler (1999) suggested that values >0.95 for the CFI and TLI, values < 0.06 for the RMSEA, and values < 0.08 for the SRMR indicate good overall model fit. Though these are not intended to be hard-and-fast cutoff values, they can be used to guide interpretation of model fit. We also used two model fit indicators to examine the incremental changes in model fit across the longitudinal MI analyses, including change in the comparative fit index (ΔCFI) and the Tucker-Lewis index (ΔTLI); when ΔCFI ≤ 0.02 (Cheung and Rensvold, 2002) and ΔTLI ≤ 0.05 (Little, 1997), then the two comparative models are not substantially different from one another. Measurement invariance researchers suggest examining a variety of overall and incremental indicators when interpreting model fit and change (Vandenberg and Lance, 2000).

Following the longitudinal MI analyses, we conducted multigroup comparisons to examine whether the MI assumptions held across genders and ethnic groups. We investigated three different ethnic groups: Black, Hispanic, and White, and all three measurement occasions were accounted for in these MI analyses.

## Results

The current data featured a nested structure (students nested within classrooms). This nesting structure was accounted for using the TYPE = COMPLEX routine in Mplus version 6.11 with the robust standard error estimator (Muthén and Muthén, 2010). Overall, 6.39% of the data were missing and had properties in line with the missing at random (MAR) condition according to missing data analyses. Therefore, participants with scores on at least one assessment wave were included in the analysis, and missing data were handled using multiple imputation. Ten datasets were imputed and the results described in the current paper represent the overall results pooled across all 10 imputations.

### Longitudinal MI

Results indicated the revised model of the engagement measure featuring three latent constructs and three sets of correlated items adequately fit the longitudinal data, $\chi_{\left(294\right)}^{2}$ = 705.406, *p* < 0.001, CFI = 0.942, TLI = 0.929, RMSEA = 0.052, SRMR = 0.052. These values are also shown in Table 6 as Model 1.1. This indicates the configural invariance assumption held for the 3-year data; all three time points had the same pattern of factor coefficients.

**Table 6:** Model fit test statistics, fit indices, and their changes for the longitudinal MI analyses of years 7, 8, and 9.

	**Model fit test statistics and fit indices**						**Change of model fit test statistics and fit indices**			
	**χ^2^**	***df***	**CFI**	**TLI**	**RMSEA**	**SRMR**	**Δχ^2^**	**Δ*df***	**ΔCFI**	**ΔTLI**
**CONFIGURAL INVARIANCE**										
1.1	705.406	294	0.942	0.929	0.052	0.052	–	–	–	–
**METRIC INVARIANCE**										
2.1 **Λ_7_ = Λ_8_**	722.772	307	0.941	0.931	0.051	0.053	**17.366**	13	**0.001**	**−0.002**
2.2 **Λ_7_ = Λ_8_ = Λ_9_**	739.208	320	0.941	0.934	0.050	0.056	**16.436**	13	**<0.001**	**−0.003**
**SCALAR INVARIANCE**										
3.1 **τ_7_ = τ_8_**	773.840	333	0.938	0.933	0.050	0.058	34.632	13	**0.003**	**0.001**
3.2 **τ_7_ = τ_8_ = τ_9_**	817.059	346	0.934	0.931	0.051	0.060	43.219	13	**0.004**	**0.002**
**FACTOR COVARIANCES INVARIANT**										
4.1 **Φ_7_ = Φ_8_**	827.766	349	0.932	0.930	0.051	0.062	10.707	3	**0.002**	**0.001**
4.2 **Φ_7_ = Φ_8_ = Φ_9_**	833.481	352	0.932	0.931	0.051	0.064	**5.715**	3	**<0.001**	**−0.001**
**FACTOR MEANS INVARIANT**										
5.1 μ_7_ = μ_8_	844.293	355	0.931	0.930	0.051	0.064	10.812	3	**0.001**	**0.001**
5.2 μ_7_ = μ_8_ = μ_9_	862.853	358	0.929	0.928	0.052	0.066	18.56	3	**0.002**	**0.002**

*Bold font indicates the difference between two comparative models is statistically negligible. Λ, factor loading matrix; τ, item intercept vector; Φ, factor covariance matrix; μ, factor mean vector; subscripts indicate the years measurements were collected; df, degrees of freedom; CFI, comparative fit index; TLI, Tucker-Lewis index; RMSEA, root mean square error of approximation; SRMR, standardized root mean square residual*.

Results of the metric longitudinal MI tests are shown in Table 6 as Model 2.1 and Model 2.2. Model fit indices indicate both models fit the data adequately. Furthermore, all model change statistics were smaller than the suggested critical values, indicating that differences in fit between the two models were statistically negligible. Since Model 2.2, the more restricted model that assumed the pattern of factor loadings was identical across time points, fit the data just as well as previous models, metric invariance was established for the engagement measure.

Results of the scalar longitudinal MI analyses are also shown in Table 6 as Model 3.1 and Model 3.2. As with previous models, fit statistics indicate both models fit the data adequately. While the Δχ^2^ model fit test statistics were statistically significant for both models, the ΔCFI and ΔTLI both indicate the models fit the data just as well as the previous models. Therefore, the assumption of scalar invariance held.

Results of the invariance analyses for the factor covariances are shown in Table 6 as Model 4.1 and 4.2. Similar to previous models, fit statistics show that both models fit the data adequately; both the ΔCFI and ΔTLI indicate that these models fit the data as well as the previous models, suggesting that covariances among the three factors are equivalent across time points. Factor correlations across all three time points for Model 4.2 are shown in Table 7. Please note that, although the covariances between the latent constructs were equivalent across time, these correlations differ slightly across all three time points because the latent factors featured slightly different variances.

**Table 7:** Factor correlations across 3 years for model 4.2.

	**BehEng with BehDis**	**BehEng with EmoEng**	**BehDis with EmoEng**
Year 7	0.798	0.524	0.678
Year 8	0.754	0.580	0.732
Year 9	0.775	0.523	0.711

*BehEng, Behavioral Engagement factor; BehDis, Behavioral Disaffection factor; EmoEng, Emotional Engagement factor. All correlations were statistically significant at the p < 0.001 level*.

Lastly, results of the invariance analyses for the factor means are also shown in Table 6 as Model 5.1 and Model 5.2. As with previous models, fit statistics indicate that both models adequately fit the data, and the ΔCFI and ΔTLI both suggest these models fit the data as well as previous models. Therefore, the means of the latent factors were assumed to be equal across time points. In sum, the longitudinal MI analyses indicated the engagement measure featured configural, metric, and scalar invariance, as well as equivalence of latent factor covariances and means across all three time points.

### Measurement invariance across gender and ethnicity

We also used MI testing procedures to examine the MI of the engagement measure across gender and ethnic groups; results of these analyses are shown in Table 8. Note that all three measurement occasions were accounted for in these analyses. For gender, models examining configural, metric, and scalar invariance, and invariance of factor covariances and means, all fit the data adequately based on model fit indices. Furthermore, although most Δχ^2^ tests indicated certain models were statistically significantly different from one another in terms of overall model fit, the ΔCFI and ΔTLI both indicated the more constrained models all fit the data just as well as the previous models. Two Δχ^2^ tests produced negative values, indicating that these difference tests cannot be interpreted and used to test for statistically significant differences in model fit. Therefore, we used the Wald test of parameter constraints to examine differences in model fit for these two comparisons. The model featuring metric invariance did not statistically significantly differ from the model featuring configural invariance in terms of overall fit, Wald $\chi_{\left(13\right)}^{2}$ = 8.484, *p* = 0.811, nor did the model featuring invariant factor covariances differ from the model featuring scalar invariance, Wald $\chi_{\left(3\right)}^{2}$ = 3.102, *p* = 0.376. In sum, based on the overall fit statistics and the ΔCFI and ΔTLI, we concluded the engagement measure featured adequate configural, metric, and scalar invariance, as well as equivalence of factor covariances and means across males and females.

**Table 8:** Model fit test statistics, fit indices, and their changes for the MI analyses for gender and ethnicity.

	**Model fit test statistics and fit indices**						**Change of model fit test statistics and fit indices**			
	**χ^2^**	**df**	**CFI**	**TLI**	**RMSEA**	**SRMR**	**Δχ^2^**	**Δdf**	**ΔCFI**	**ΔTLI**
**GENDER**										
Configural	585.500	196	0.942	0.929	0.050	0.050	–	–	–	–
Metric **Λ**_M_ = **Λ**_F_	585.024	209	0.944	0.935	0.048	0.052	−0.476^*^	13	**−0.002**	**−0.006**
Scalar **τ**_M_ = **τ**_F_	630.885	222	0.939	0.934	0.048	0.053	45.861	13	**0.005**	**0.001**
Factor Covariances Φ_M_ = Φ_F_	630.323	225	0.939	0.935	0.048	0.058	−0.562^*^	3	**<0.001**	**−0.001**
Factor Means **μ**_M_ = **μ**_F_	650.970	228	0.937	0.933	0.049	0.070	20.647	3	**0.002**	**0.002**
**ETHNICITY**										
Configural	648.856	294	0.946	0.934	0.049	0.051	–	–	–	–
Metric Λ_B_ = Λ_H_ = Λ_W_	682.103	320	0.945	0.938	0.047	0.060	33.247	26	**0.001**	**−0.004**
Scalar τ_B_ = τ_H_ = τ_W_	781.222	346	0.934	0.931	0.050	0.066	99.199	26	**0.011**	**0.007**
Factor Covariances **Φ**_B_ = **Φ**_H_ = **Φ**_W_	789.592	352	0.934	0.932	0.050	0.073	**8.37**	6	**<0.001**	**−0.001**
Factor Means μ_B_ = μ_H_ = μ_W_	832.159	358	0.928	0.928	0.051	0.084	42.567	6	**0.006**	**0.004**

Bold font indicates the difference between two comparative models is statistically negligible. Λ, factor loading matrix; τ, item intercept vector; Φ, factor covariance matrix; μ, factor mean vector; subscripts indicate the groups of the measures collected; df, degrees of freedom; CFI, comparative fit index; TLI, Tucker-Lewis index; RMSEA, root mean square error of approximation; SRMR, standardized root mean square residual; M, male; F, female; B, Black; H, Hispanic; W, White.

* *Denotes the negative test statistic cannot be interpreted, and a Wald test of parameter constraints was examined instead*.

Regarding ethnicity, model fit indices suggest the five models testing the configural, metric, and scalar invariance, and invariance of latent factor covariances and means, of the engagement measure all fit the data reasonably well (see Table 8). Although some Δχ^2^ tests suggested that some models fit the data statistically significantly differently from one another, the ΔCFI and ΔTLI both indicated that the more constrained models fit the data as well as the previous models that feature fewer model constraints. Thus, the configural, metric, and scalar invariance assumptions held across ethnic groups, as did invariance of factor covariances and means.

## Discussion

### Engagement measure MI

The current results indicated that the classroom engagement measure featured adequate configural, metric, and scalar invariance across time points, genders, and ethnicities. These results suggest that scores on the engagement measure have approximately the same meaning across these groups, and that this measure is appropriate for use when testing substantive hypotheses regarding developmental changes between grades 7 and 9, as well as gender and ethnic differences in engagement among students within this grade range.

The present results tie in well with previous literature examining the MI of other classroom engagement measures. Existing research has shown that other measures based on different theoretical conceptualizations of engagement are also invariant across groups. For example, engagement measures featuring cognitive, affective, and behavioral components were found to be invariant across genders and ethnic groups (Glanville and Wildhagen, 2007; Wang et al., 2011). Research on more complex engagement measures, such as Maryland's Safe and Supportive Schools Initiative Survey (Bradshaw et al., 2014) and the Motivation and Engagement Scale (Marsh et al., 2011), have also demonstrated these measures' invariance across various groups such as genders and grade levels. The current study's findings add to the psychometric literature on classroom engagement measures, demonstrating that this measure, which is based on Skinner et al. (2008) theoretical conceptualization of engagement, is also invariant across grade levels, ethnic groups, and genders. Therefore, the engagement measure examined in the current study is an additional measure, stemming from a different theoretical perspective on engagement that may be used in substantive research to explore cross-group comparisons in behavioral engagement, behavioral disaffection, and emotional engagement.

Furthermore, the current findings also indicated that the factor means and covariances were invariant across time points, ethnic groups, and genders, suggesting that average levels of behavioral engagement, behavioral disaffection, emotional engagement, and the relationships between them remained consistent across these groups. The current findings align with those from previous research regarding factor covariances; existing research has shown that relationships among behavioral engagement and disaffection, and emotional engagement were invariant across grade levels (Skinner et al., 2008, 2009) and genders (Skinner et al., 2009). However, the results regarding invariant factor means run counter to those observed in prior engagement research.

Regarding grade level-related changes in engagement, research on elementary and middle school students has shown that elementary students tend to have higher emotional and behavioral engagement than middle school students (Skinner et al., 2008, 2009); additional research suggests that classroom disengagement increases between elementary and middle school (Wang et al., 2014). That said, the lack of change in engagement across grade levels in the current study may be because changes in classroom engagement occur primarily between elementary and middle school. In past research, changes in classroom engagement have been attributed to younger children being developmentally different than older children in terms their learning strategies, self-regulation, and other cognitive factors related to classroom engagement (Fredricks and McColskey, 2012; Sinatra et al., 2015). Because the current student sample is older than those examined in prior research, engagement levels may have stabilized by the time students reached grade 7 and remained consistent throughout grades 7, 8, and 9.

Regarding gender, previous research indicates that girls tend to be higher in behavioral and emotional engagement than boys (Skinner et al., 2008, 2009; Wang et al., 2011, 2014); Wang and Fredricks (2014) also observed that girls were higher in cognitive engagement than boys. It is unknown at this time why the current results do not align with those from previous research. However, due to the nature of the current sample being composed of lower-achieving students, there may have been additional variables at play that impacted the present findings that may not otherwise be present in other samples. Previous research has shown that outside variables, such as teacher-student interaction quality (Rimm-Kaufman et al., 2015) do interact with how gender relates to engagement; it is possible that such variables played a role in the current study, but were not accounted for.

Lastly, prior research on ethnicity suggests that White students tend to have higher behavioral engagement and lower emotional engagement than Black students (Wang et al., 2011; Wang and Fredricks, 2014). Once again, it is unknown why the current results do not match those from past studies. As with the issue described above regarding the lack of gender differences, other variables associated with the low-achieving sample makeup may have played a role in the current results.

### Limitations and future directions

One limitation of the current study is that the sample was selected based on students who scored below the median on a district-administered literacy measure. Therefore, the current results may apply only to lower-achieving students and not to normally- or higher-achieving students. A second limitation was that the engagement measure was administered at four different time points, only three of which were used in the current study. Although the current analyses indicated the measurement of the classroom engagement measure was consistent across the 3-year period, because it was not administered at a larger number of time points, it is impossible to know how well the longitudinal MI would hold over a longer period of time. Future research is needed to examine the measurement properties of this engagement measure in both more diverse samples, and over longer periods of time.

Furthermore, the three-factor CFA model that was examined features some caveats that should be accounted for when using and interpreting this engagement measure. First, the Emotional Engagement subscale featured relatively poor internal consistency reliability compared to the other two subscales (see Table 2). Although Emotional Engagement's internal consistency was low in this particular study, reliability estimates can vary between different samples and test administrations (Henson, 2001). Therefore, the low reliability observed in the current study may be due to the nature of the sample that was studied. Future researchers employing this classroom engagement measure should examine the reliability of all three subscales, which would help identify whether the low internal consistency in the current study was an anomaly or part of a broader pattern. Also, although the current CFA model fit the data well overall, it featured only moderate convergent and discriminant validity. Future researchers should bear in mind these slight validity limitations and take note that the engagement subscales are highly related to one another, as shown both in the current study, and in previous research (Skinner et al., 2009).

## Conclusion

The three-factor engagement measure examined in the current study, derived from Skinner et al.'s (2008) theoretical conceptualization, features adequate configural, metric, and scalar MI, as well as equivalence of factor covariances and means, across grade levels, genders, and ethnicities; our results support the psychometric consistency of the engagement measure across these three variables.

Therefore, based on the current study's findings, this measure of classroom engagement is suitable for testing hypotheses regarding group differences in engagement across genders and ethnicities, as well as for studying grade level-related changes in engagement. Given the stability of this measure across genders, ethnicities, and grades, researchers may also use it to examine relationships between the engagement factors and other important academic outcomes.

## Author contributions

RG: conducted the data analyses and interpretation, performed majority of the writing, and edited the final paper. QC: developed the original research idea, gained access to the data, and guided the overall research process.

### Conflict of interest statement

The authors declare that the research was conducted in the absence of any commercial or financial relationships that could be construed as a potential conflict of interest.

## References

[B1] Bradshaw C. P. Waasdorp T. E. Debnam K. J. Johnson S. L. (2014). Measuring school climate in high schools: a focus on safety, engagement, and the environment. J. Sch. Health 84, 593–604. 10.1111/josh.12186 25117894

[B2] Burch G. F. Heller N. A. Burch J. J. Freed R. Steed S. A. (2015). Student engagement: developing a conceptual framework and survey instrument. J. Educ. Bus. 90, 224–229. 10.1080/08832323.2015.1019821

[B3] Byrne B. M. (1998). Structural Equation Modeling with LISREL, PRELIS, and SIMPLIS: Basic Concepts, Applications, and Programming. Mahwah, NJ: Lawrence Erlbaum.

[B4] Chen Q. Hughes J. N. Liew J. Kwok O. (2010). Joint contributions of peer acceptance and peer academic reputation to achievement in academically at-risk children: mediating processes. J. Appl. Dev. Psychol. 31, 448–459. 10.1016/j.appdev.2010.09.001 21113406 PMC2992319

[B5] Cheung G. W. Rensvold R. B. (2002). Evaluating goodness-of-fit indexes for testing measurement invariance. Struct. Equ. Model. 9, 233–255. 10.1207/S15328007SEM0902_5

[B6] Fredricks J. A. McColskey W. (2012). The measurement of student engagement: A comparative analysis of various methods and student self-report instruments, in Handbook of Research on Student Engagement, eds Christenson S. L. Reschly A. L. Wylie C. (New York, NY: Springer), 763–782.

[B7] Glanville J. L. Wildhagen T. (2007). The measurement of school engagement: assessing dimensionality and measurement invariance across race and ethnicity. Educ. Psychol. Meas. 67, 1019–1041. 10.1177/0013164406299126

[B8] Henson R. K. (2001). Understanding internal consistency reliability estimates: a conceptual primer on coefficient alpha. Meas. Eval. Counsel. Dev. 34, 177–189.

[B9] Hu L. Bentler P. M. (1999). Cutoff criteria for fit indexes in covariance structure analysis: conventional criteria versus new alternatives. Struc. Equ. Model. 6, 1–55. 10.1080/10705519909540118

[B10] Kline R. B. (2010). Principles and Practice of Structural Equation Modeling, 3rd Edn. New York, NY: Guilford.

[B11] Lee J. (2014). The relationship between student engagement and academic performance: is it a myth of reality? J. Educ. Res. 107, 177–185. 10.1080/00220671.2013.807491

[B12] Little T. D. (1997). Mean and covariance structures (MACS) analyses of cross-cultural data: practical and theoretical issues. Multivariate Behav. Res. 32, 53–76. 10.1207/s15327906mbr3201_3 26751106

[B13] Lutz Klauda S. Guthrie J. T. (2015). Comparing relations of motivation, engagement, and achievement among struggling and advanced adolescent readers. Read. Writ. 28, 239–269. 10.1007/s11145-014-9523-2 25663747 PMC4314949

[B14] Marsh H. W. Liem G. A. D. Martin A. J. Morin A. J. S. Nagengast B. (2011). Methodological measurement fruitfulness of exploratory structural equation modeling (ESESM): new approaches to key substantive issues in motivation and engagement. J. Psychol. Assess. 29, 322–346. 10.1177/0734282911406657

[B15] Martin A. J. Yu K. Papworth B. Ginns P. Collie R. J. (2015). Motivation and engagement in the United States, Canada, United Kingdom, Australia, and China: testing a multi-dimensional framework. J. Psychol. Assess. 32, 103–114. 10.1177/0734282914546287

[B16] Millsap R. E. (2011). Statistical Approaches to Measurement Invariance. New York, NY: Routledge.

[B17] Muthén L. K. Muthén B. O. (2010). Mplus User's Guide, 6th Edn. Los Angeles, CA: Muthén and Muthén.

[B18] Rimm-Kaufman S. E. Baroody A. E. Larsen R. A. A. Curby T. W. Abry T. (2015). To what extend do teacher-student interaction quality and student gender contribute to fifth graders' engagement in mathematics learning? J. Educ. Psychol. 107, 170–185. 10.1037/a0037252

[B19] Sinatra G. M. Heddy B. C. Lombardi D. (2015). The challenges of defining and measuring student engagement in science. Educ. Psychol. 50, 1–13. 10.1080/00461520.2014.1002924

[B20] Skinner E. A. Wellborn J. G. Connell J. P. (1990). What it takes to do well in school and whether I've got it: a process model of perceived control and children's engagement and achievement in school. J. Educ. Psychol. 82, 22–32.

[B21] Skinner E. A. Zimmer-Gembeck M. J. Connell J. P. (1998). Individual differences and the development of perceived control. Monogr. Soc. Res. Child Dev. 63 i–vi, 1–220. 10.2307/1166220 9839459

[B22] Skinner E. Furrer C. Marchand G. Kindermann T. (2008). Engagement and disaffection in the classroom: Part of a larger motivational dynamic? J. Educ. Psychol. 100, 765–781. 10.1037/a0012840

[B23] Skinner E. Kindermann T. A. Furrer C. J. (2009). A motivational perspective on engagement and disaffection: conceptualization and assessment of children's behavioral and emotional participation in academic activities in the classroom. Educ. Psychol. Meas. 69, 493–525. 10.1177/0013164408323233

[B24] Vandenberg R. J. Lance C. E. (2000). A review and synthesis of the measurement invariance literature: suggestions, practices, and recommendations for organizational research. Organ. Res. Methods 3, 4–70. 10.1177/109442810031002

[B25] Wang Z. Bergin C. Bergin D. A. (2014). Measuring engagement in fourth to twelfth grade classrooms: the classroom engagement inventory. Sch. Psychol. Q. 29, 517–535. 10.1037/spq0000050 24708283

[B26] Wang M. T. Fredricks J. A. (2014). The reciprocal links between school engagement, youth problem behaviors, and school dropout during adolescence. Child Dev. 85, 722–737. 10.1111/cdev.12138 23895361 PMC3815520

[B27] Wang M. T. Willett J. B. Eccles J. S. (2011). The assessment of school engagement: examining dimensionality and measurement invariance by gender and race/ethnicity. J. Sch. Psychol. 49, 465–480. 10.1016/j.jsp.2011.04.001 21724001

[B28] Woodcock R. W. McGrew K. S. Mather N. (2001). WJ-III Tests of Achievement. Itasca, IL: Riverside.

[B29] Wu J.-Y. Hughes J. N. (2015). Teacher Network of Relationships Inventory: measurement invariance of academically at-risk students across ages 6 to 15. School Psychol. Q. 30, 23–36. 10.1037/spq0000063 24884450 PMC4254382

[B30] Wu J. Y. Hughes J. N. Kwok O. M. (2010). Teacher-student relationship quality type in elementary grades: effects on trajectories for achievement and engagement. J. Sch. Psychol. 48, 357–387. 10.1016/j.jsp.2010.06.004 20728688 PMC2928164

